# Application of metabolomics in urolithiasis: the discovery and usage of succinate

**DOI:** 10.1038/s41392-023-01311-z

**Published:** 2023-01-21

**Authors:** Xiu-zhen Zhang, Xiong-xin Lei, Yan-lin Jiang, Long-mei Zhao, Chen-yu Zou, Yun-jin Bai, Ya-xing Li, Rui Wang, Qian-jin Li, Qiu-zhu Chen, Ming-hui Fan, Yu-ting Song, Wen-qian Zhang, Yi Zhang, Jesse Li-Ling, Hui-qi Xie

**Affiliations:** 1 Laboratory of Stem Cell and Tissue Engineering, Orthopedic Research Institute, Department of Orthopedics, State Key Laboratory of Biotherapy, West China Hospital, Sichuan University, Chengdu, Sichuan 610041 China; 2 Department of Urology, Institute of Urology, West China Hospital, Sichuan University, Chengdu, Sichuan 610041 China; 3 Research Core Facility of West China Hospital, Sichuan University, Chengdu, Sichuan 610041 China; 4 Department of Medical Genetics, West China Second University Hospital, Chengdu, Sichuan 610041 China

**Keywords:** Metabolic disorders, Drug development

## Abstract

Urinary stone is conceptualized as a chronic metabolic disorder punctuated by symptomatic stone events. It has been shown that the occurrence of calcium oxalate monohydrate (COM) during stone formation is regulated by crystal growth modifiers. Although crystallization inhibitors have been recognized as a therapeutic modality for decades, limited progress has been made in the discovery of effective modifiers to intervene with stone disease. In this study, we have used metabolomics technologies, a powerful approach to identify biomarkers by screening the urine components of the dynamic progression in a bladder stone model. By in-depth mining and analysis of metabolomics data, we have screened five differential metabolites. Through density functional theory studies and bulk crystallization, we found that three of them (salicyluric, gentisic acid and succinate) could effectively inhibit nucleation in vitro. We thereby assessed the impact of the inhibitors with an EG-induced rat model for kidney stones. Notably, succinate, a key player in the tricarboxylic acid cycle, could decrease kidney calcium deposition and injury in the model. Transcriptomic analysis further showed that the protective effect of succinate was mainly through anti-inflammation, inhibition of cell adhesion and osteogenic differentiation. These findings indicated that succinate may provide a new therapeutic option for urinary stones.

## Introduction

Urolithiasis is a relatively typical urological disorder whose worldwide prevalence has continuous increased during the past few decades.^1^ It is also a disease with a 5-year relapse rate as high as 50%.^2^ The etiology of urolithiasis is complex and may be influenced by obesity, diabetes, hypertension and metabolic syndromes.^3^ Calcium oxalate (CaOx) stones have accounted for over 75% of urinary stones, which is followed by uric acid, calcium phosphate, struvite, and cystine types.^4^ Urolithiasis is a multi-step process involving crystal nucleation, growth and aggregation, though the underlying mechanism is not yet fully understood.^5^ How to inhibit urolithiasis and predict its underlying risk, remained a great challenge.^6^

The heterogeneity of stone types among patients create requirement for personalized treatment management, which may include dietary regulation/water intake, administration of several kind of FDA-approved drugs, and surgical procedure to remove the stones.^7^ Nowadays, the problems about urolithiasis recurrence and prevalence still raised great socioeconomic attention. Therefore, to seek new treatments is necessary.^8^

Along with the seeking of medicine for the prevention and treatment of calculi, modifiers which can influence crystallization have attracted increased attention.^9^ The most efficient modifiers of calcium stones are composed of carboxylic acid, sulfate function groups which can interact with crystal surface and/or complex with free ions in the urine.^10^ Many natural and synthetic species can inhibit its growth. But it is still very hard to bridge the in vitro and in vivo evidence. The combination of experimental study, animal studies and clinical trials may facilitate our cognition on crystal growth modification and its function in crystallization.^5^ To date, only two molecules, i.e., polyacrylic acid and a divalent inositol phosphate molecule,^10,11^ have shown to inhibit CaOx crystallization by reducing renal CaOx deposition.

The pathogenesis of urolithiasis associated with many metabolic and outer factors. Although the mechanism is not yet fully understood, a fact is undoubtedly that it is influenced by urine composition.^4^ Urine is an kind of complex mixture of abundant molecular species including both inhibitors and promoters for the development of kidney stones.^12^ Nephrolithiasis has a high occurrence in spinal bifida patients who had suffer bladder stones after the augmentation.^13^ Metabolic disorder is closely related with noninfectious stones.^14^ To assess the inhibitors unique effect(s) is very hard for urine complexity, which include ~3100 small molecule metabolites, and half of which has not be identified. Urine contains about 1823 proteins, among which 671 are recently identified constituents.^15^

Metabonomics mainly studies small molecular substances in cells and tissues. These substances are closely related to the health of the body and mainly obtained through metabolism of various substances under physiological or pathological conditions. At present, in the field of personalized therapy, small molecule substances begin to receive extensive attention, and become the cornerstone of biomarker research.^16,17^ In the field of kidney research, metabonomics has been widely used and has achieved remarkable results, and has also been used to study new markers for the diagnosis of kidney diseases.^18^ With the rapid development of bio-information technology, the application scope of metabonomics technology is also expanding. For example, it has been applied in the field of disease mechanism research and target selection. In addition, metabonomics methods also have high application value in CaOx stone animal model research field, through which many abnormal metabolic pathways were identified.^19^ Recently, metabolomics approach has also been used to investigate the urine of patients with kidney stones, which may be of high value for the therapy of this disease.^20^

Our previous studies have shown that the calculi will always form in the rabbit bladder in the early period (1~2 weeks) after the bladder augmentation, and may either resolve or develop over time.^21^ The disappearance of urinary stone was puzzled as the calculi was much bigger than the circumference of the rabbit urethra, and it was difficult to excrete autonomic, until a recent study on CaOx crystals has found a new inhibition pathway which can lead to stone dissolution with very few modifiers.^22^ To identify the new crystal inhibitors, we have analyzed the urine samples from both bladder stone model and normal control by using untargeted metabolomics, with an aim to identify novel targets for therapeutic intervention and bridge the in vitro and in vivo discoveries.

## Results

### The dynamic change of urinary bladder stone and its component

**Fig. 1:**
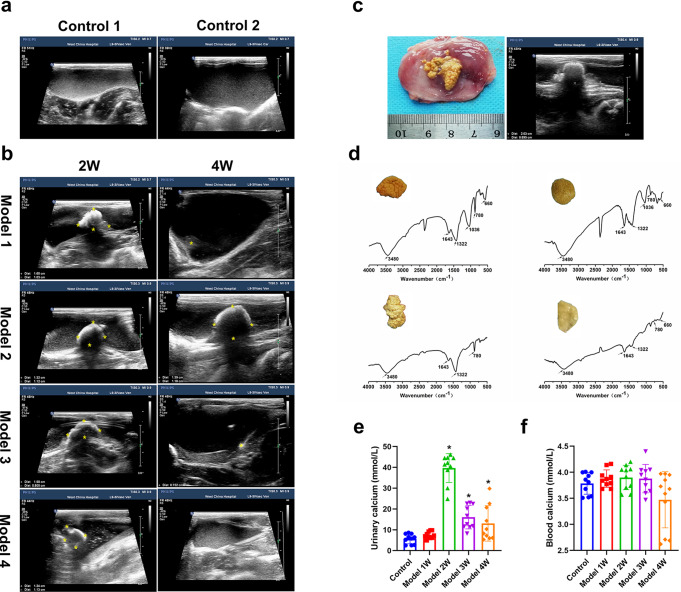
The dynamic changes of stone and component analysis. **a**, **b** Representative images of the bladder by abdomen ultrasound of normal control and bladder stone model at 2 and 4 weeks. **c** Gross view and abdomen ultrasound of the stone sample from the same individual. **d** Infrared spectra and gross morphology of the stone. **e**, **f** Quantification of the calcium level in the urine and blood samples. *P < 0.05 compared with control group. For (**e**–**f**), *n* = 10 rabbits in each group

We have used the PC-SIS (Procyanidins crosslinked small intestine submucosa) to repair the full-thickness bladder defects in a rabbit model for bladder stone.^21^ Ultrasonography examination revealed strong echo and shadow in the lumen of urinary bladder of all model animals after 2 weeks (Fig. 1b). The calculus harvested to visualize the size, distribution and component. From the Fig. 1c, d, the calculi were irregular in shape with a texture beige surface, whilst the result abdominal ultrasound had closely matched with the gross appearance of the calculi. By FTIR spectrum, which can provide a reliable characterization and highlight the composition of the urinary stones at atomic scale (Fig. 1d), CaOx and hydroxyapatite (HAP) are typical components of the samples. The absorption bands at 1036 cm^−1^ are due to HAP stretching, whereas that at 660 cm^−1^ may corresponding to the COM vibrations. The diagnostic bands of 1643 cm^−1^ (C = O vibration), and 780 cm^−1^ (C-C stretching) are characteristic of Calcium. As discovered, calcium oxalate dihydrate (COD) has made a significant contribution to the sample.^23^ By biochemical urinalysis (Fig. 1e, f), the level of calcium has significantly increased 2 weeks after the surgery. By contrast, the concentration of blood calcium did not differ between the model animal and the normal controls.

Notably, up to 70% of the stones have disappeared by 4 weeks (Fig. 1b). The urethral size of adult rabbits varies from 4 to 10 mm under different urethral pressures, which is much lower than the size of the stone, it is difficult to be excreted spontaneously (Supplementary Table 1).^24^ The formation of urinary stones undergo multiple processes which contains crystal nucleation, crystal formation and aggregation, with the latter regulated by crystal inhibitors and promoters.^25^ Microscopy analysis showed evidence that the dissolution has mainly occurred during the minerogenetic process of kidney stones.^26^ This has contradicted the widely held concept that the stones cannot dissolve,^27,28^ and opened a new paradigm for clinical approaches, where targeted in vivo dissolution can alleviate the harmful influence of the stone disease. Meanwhile, this has also led us to reflect whether the dynamic changes of calculus may be attributed to the presence of crystal modifiers in urine. As an intricate mixture of metabolites, the urine contains ~3100 small molecules with some active modifiers (crystal inhibitors as well as promoters).^29^ As such, to identify the inhibitors which may influence the pathological stone development is difficult.

### Metabolomic profiling of urine samples

**Fig. 2:**
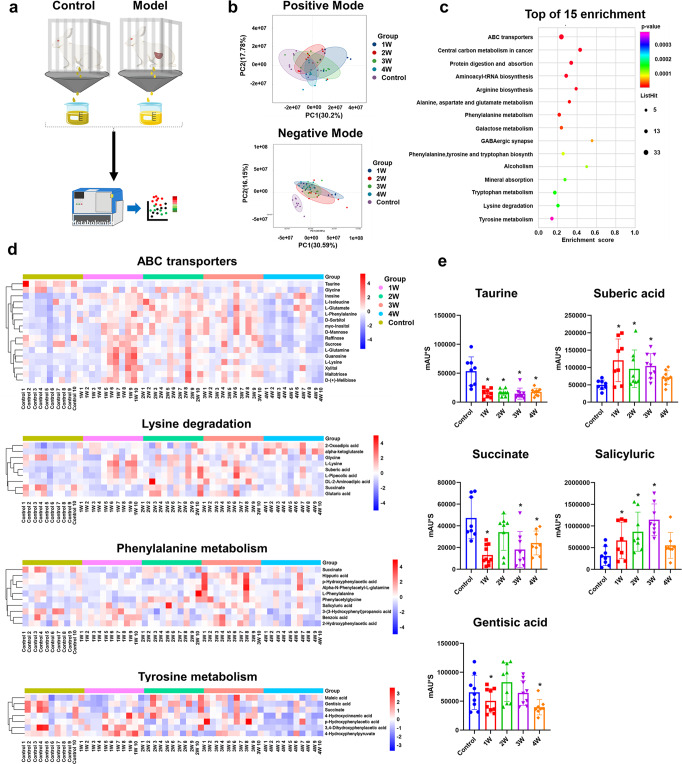
Metabolomics profiling has identified five organic molecules as the candidate for crystal inhibitors. **a** Schematic representing the design of metabolomics profiling experiments. **b** Principal coordinate analysis (PCoA) for the positive and negative ions of the control and model animal based on metabolite profiles of the urine samples. **c** KEGG pathway enrichment analysis of the control and model groups. **d** Heatmap of ABC transporters, lysine degradation, phenylalanine metabolism and tyrosine metabolism pathway. **e** Alteration of the five organic molecules based on metabolite profiles and statistical analysis, *P < 0.05 compared with control group. For (**a**–**e**), *n* = 10 rabbits in each group

The likely presence of crystal modifiers suggested that some natural urinary compositions, including metabolites, may affect the crystal growth and stone formation.^29^ To further investigate the unique effect(s) of inhibitors, we have carried out an untargeted metabolomics analysis on 50 independent urine samples from normal controls (10 samples) and bladder stone models (10 samples) at different time points (1, 2, 3 and 4 weeks after the bladder augmentation surgery) (Fig. 2a). From the Fig. 2b, negative metabolites were clearly separated between the normal control and surgery-treated groups based on principal coordinate analysis (PCoA). For this purpose, we have focused on the negative metabolites, and quantified the segregation between the control and the model at various time points after surgery by PLS-DA analysis, and discovered a obvious difference between the two profiles (Supplementary Fig. 1). Pathway analysis by pair-wise (Supplementary Fig. 1) and multiple group comparisons (Fig. 2c) have identified the ABC transporters, lysine degradation, phenylalanine metabolism and tyrosine metabolism pathway as the significant enrichment in the KEGG. To further delineate the metabolites involved in the enriched pathways, we have plotted the heatmaps of the control and model groups for the four pathways (Fig. 2d and Supplementary Fig. 1) to reveal the difference in metabolites between the two groups.

Polyprotic organics and crystal can interact by electrostatics in the calcium mineralization system, where the criteria for screening an effective inhibitor is a molecule possessing multiple charged groups.^30^ In fact, some of the most effective inhibitors for calcium crystal had carboxylate (–COOH), hydroxyl (–OH) and/or sulfate (–OSO^3^–) functional groups which can interact with crystal surface in urine.^31,32^ We therefore selected five molecules with functional groups based on the heatmaps analysis, which included Taurine (–OH), Suberic acid (–COOH), Succinate (–COOH), Salicyluric (–COOH, –OH) and Gentisic acid (–COOH, –OH) from the differentially expressed metabolites for further analysis (Fig. 2e). Statistical analysis showed that the change of organic acid level did not reveal any obvious regularity, suggesting that they have played diverse roles in crystal modification.

### In vitro experiments for CaOx inhibition

**Fig. 3:**
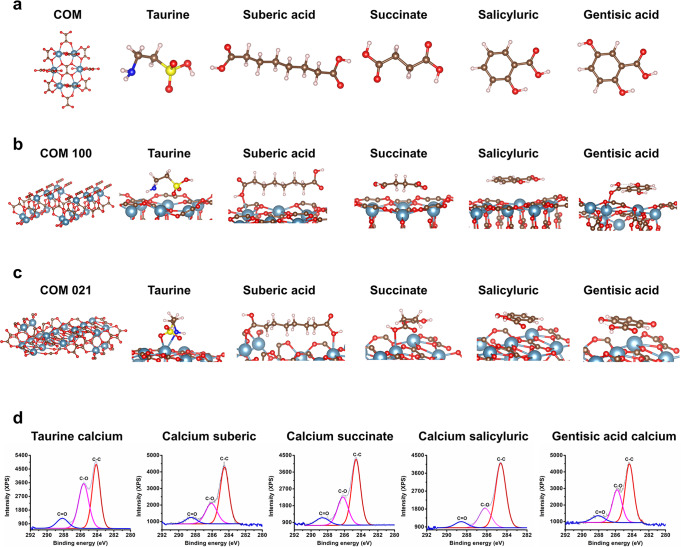
Binding energy of the modifiers on the COM surface and Ca^2+^. **a** The optimized structures of COM, taurine, suberic acid, succinic, salicyluric and gentisic acids. **b**, **c** Optimized structures of the molecules on the COM (100) and COM (021) surface, with the light-pink, light-blue, brown, blue, yellow and red balls denoting the H, Ca, C, N, S and O atoms, respectively. **d** X-ray photoelectron spectra (XPS) of taurine, suberic acid, succinic, salicyluric and gentisic acids adsorbed to Ca^2+^. The fitted peaks in blue, pink and red correspond to the C1s peaks of C = O, C–O and C–C, respectively

Density functional theory (DFT) was used to shed light on the action of the modifiers. The adsorption energy of the modifier on the COM (100) surface was gentisic acid > salicyluric > succinic acid > suberic acid > taurine. The binding energies of the modifiers on the COM (021) surface were in keeping with those of the COM (100) surface except for salicyluric and suberic acid (Supplementary Table 2). Compared with other modifiers, the strength of binding between gentisic acid with the COM (100) and (021) surface (Fig. 3b, c) was greater. To quantify the strength of modifiers bound to COM (100) and (021) surfaces, we have calculated the mean displacement δ of atoms (Supplementary Table 3). The result revealed that all modifiers adsorption on the (100) surface has resulted in a higher strain compared with the (021) surface. The binding of gentisic acid with the COM surface has led to the highest strain (100 δ = 0.418 Å; 021 δ = 0.206 Å). By contrast, taurine has the lowest strain (100 δ = 0.211 Å; 021 δ = 0.099 Å). It has been previously demonstrated that under constant tensile stress, the strain of the crystal could reduce its growth rate. This also suggested that the effect of gentisic acid on preventing crystal growth is better than that of taurine.^22,33^

In addition to COM surface interactions, this study also analyzed the coordination of modifiers with Ca^2+^ (Supplementary Fig. 2a, Supplementary Table 4). The calculations indicated that gentisic acid, salicyluric and succinate displayed a higher affinity for Ca^2+^ ion complex compared with suberic acid and taurine. The binding of gentisic acid with Ca^2+^ was most energetic. The interaction types of modifiers and Ca^2+^ were detected based on XPS spectroscopy (Fig. 3d, Supplementary Fig. 2b and Table 5). The binding energy in XPS spectroscopy rest with a tiny chemical shift caused by atomic charges. Hence, it is associated with the net atomic charge.^34^ We have focused on the changes in the organic acid calcium and organic acid C1s peaks with C = O that can interact with Ca^2+^. The change range of the C = O of organic acid and organic acid calcium reflected the size of the binding energy, and the change trend as follows: gentisic acid > salicyluric > succinate > suberic acid > taurine with the same trend as DFT calculations. Considering the diversity of inhibitory effects of the modifiers, we need further demonstrated the inhibitory effect of the modifiers through in vitro and in vivo experiments.

### In vivo experiment for CaOx inhibition

**Fig. 4:**
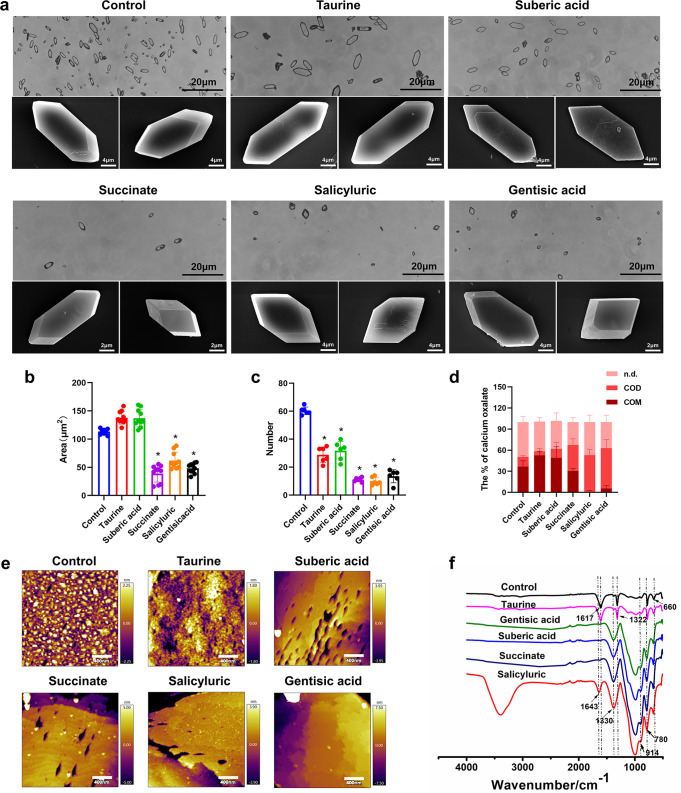
Inhibition of COM nucleation in vitro. **a** Optical (scale bar: 20 μm) and SEM (scale bar: 2 μm & 4 μm) micrographs. **b**–**d** Semi-quantitative analysis of the crystalized area and the number and ratio of COM and COD (COM—CaOx monohydrate, COD—CaOx dihydrate, n.d.—not defined), *P < 0.05 compared with the control group. **e** AFM images of (001) surface growth and inhibition with or without the modifier (scale bar: 400 nm). **f** FTIR spectra with or without the modifier

With or without modifiers, the bulk crystallization is detected through a microscope to determine the corresponding changes in crystal size and morphology. This technology does not need to conduct invasion operation when analyzing crystallization, so as to analyze the effect of modifier more efficiently and accurately. By optical micrography, the taurine and suberic acid showed no apparent effect on the crystal morphology and area compared with the control. By contrast, succinate, salicyric and gentisic acid have generated classic tetragonal-shaped crystals (Fig. 4a, b). This has reflected inhibition in crystal growth rate and underlying suppression of COM nucleation (Fig. 4b, 4c). The classical mechanism of crystal growth included 2D layered nucleation and step advanced across crystal planes. The presence of inhibitors on crystal surface can hinder the formation and growth of the crystals.^35^ Atomic force microscopy (AFM) of the crystal surface topology (Fig. 4e) showed that the control and Taurine crystals had the smallest changes in depth (within 3 nm), although there are defects on the crystal surface. Following the intervention with gentisic acid and succinate, obvious crystal growth can be seen. The surface defect of gentisic acid-treated crystals was the largest, with a range of about 8 nm. Deep regular cracks (depth: 5 nm) can be observed in succinate crystals during the steps of growth, which may be attributed to that the crystal inhibitors have changed the direction of crystal growth, and further led to decreased stability of the crystal structure.

The previous studies have mainly paid attention to the influence of inhibitors on COM crystallization, our analysis predominantly concentrated in the induction of COM and COD. To evaluate the latter, we have measured the ratio of COM. As shown in Fig. 4d, both taurine and suberic acid have significantly promoted COM nucleation (52% ± 5% and 49% ± 15%, respectively) compared with normal control (36% ± 8%). Succinate, salicyric and gentisic acid have all led to <30% of COM nucleation and >37% of COD nucleation compared with the normal control (13% ± 2%), which confirmed their inhibitory effect on COM nucleation. FTIR experiments (Fig. 4f) also revealed a shift from mostly-COM to mostly-COD crystallization with succinate, salicyric and gentisic acid in the bulk crystallization system. The absorption peak at 660 cm^−1^ in fingerprint region indicated that the bulk of crystal phase was COM. In addition, 914 and 780 cm^−1^ were absorption peaks of COD. The FTIR spectra of the taurine groups were similar, with the characteristic absorption peaks at 660 cm^−1^ indicating that the bulk of crystal phase of the CaOx crystal was COM. After adding salicyluric, suberic acid, gentisic acid and succinate, the absorption peaks of the crystal solution have shifted from 1617 and 1322 cm^−1^ to 1643 and 1330 cm^−1^, which indicated that the addition of modifiers has led to increased COD, which was consistent with characterization of the crystal morphology.^36^ CaOx crystals have two common forms: COM and COD. COD is less bound with instability, and whilst COM is more easily attached to renal epithelial cells and can aggregate to form stones.^37^ Thus, a method to suppress stone formation may be to screen modifiers which can target the CaOx crystallization as well as induce the formation of COD. Compared with the control, the increased instability of the COD crystals with the addition of succinate, salicyluric acid and gentisic acid has suggested a potential role for the treatment of kidney stones.

### Reduction of kidney CaOx deposition and injury by succinate

**Fig. 5:**
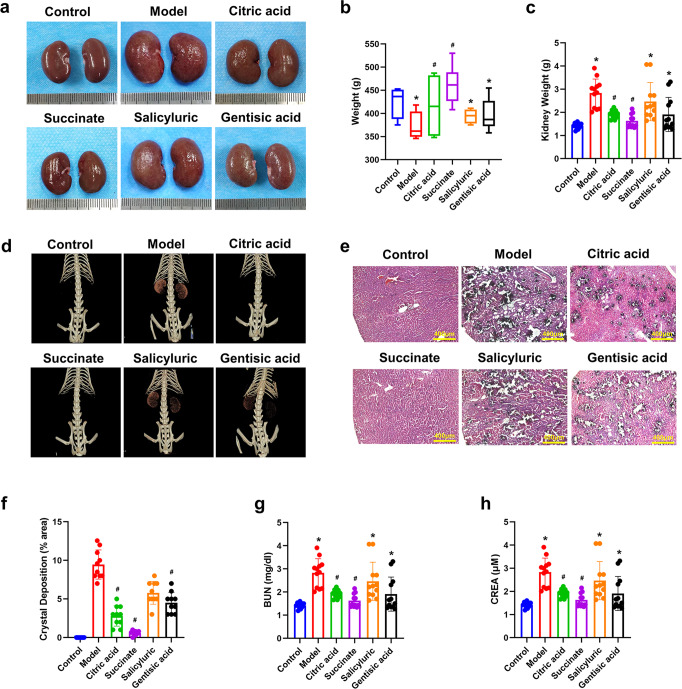
Succinate has reduced kidney crystal deposition and injury in the EG-treated rat CaOx crystallization model. **a** Gross appearance of the kidneys. **b**, **c** Body and kidney weight, *P < 0.05 compared with the control group; # *P* < 0.05 compared with the model group. **d** Micro-CT imaging, **e**, **f** Sections of rat kidney were stained for crystal deposition by Von Kossa staining (scale bar: 400 μm), and the dark areas of the crystal were quantified, # *P* < 0.05 compared with the model group. **g**, **h** Plasma blood urea nitrogen (BUN) and creatinine (CREA), *P < 0.05 compared with the control group; # *P* < 0.05 compared with the model group

We have selected modifiers with in vitro crystal inhibition (salicyluric, gentisic acid and succinate) to assess whether polyprotic organics have impact on EG-induced crystal formation. Citric acid, the main component of the stone intervention drug Ulaite, was applied as the intervention group. Sprague-Dawley rats were treated with inhibitors (200 mg/kg) or 0.9% NaCl by daily gavage. In the meantime, experimental group and model group rats accepted 1% EG solution to induce crystals formation. Four weeks later, the gross appearance (Fig. 5a) and weight (Fig. 5c) of the rat kidneys, as well as body weight (Fig. 5b) were analyzed to assess the basic condition of the rats. Both citric acid and succinate have shown a protective effect for the EG-induced rat model, the succinate intervention group had similar kidney appearance, weight and body weight with those of the normal control, whereas salicyluric and gentisic acid showed no significant effect for kidney protection. While NaCl, salicyluric and gentisic acid treated EG-induced rat models have developed stones within 4 weeks, those treated with citric acid and succinate showed significant inhibition for stone formation, along with lower level of blood BUN and plasma creatinine (Fig. 5d–h).

We have further assessed the influence of modifiers on renal functions in various groups of rats. By Von Kossa staining (Fig. 5e, f, Supplementary Fig. 3), there were abundant crystal crystals in the border area between cortex and medulla in EG-treated rats. Compared with the citric acid, salicyluric and gentisic acid, CaOx crystal deposition was obviously lower in succinate group after the EG treatment (*P* < 0.05). Kidney injury was detected by Periodic Acid-Schiff (PAS) staining and scored for tubular injury (Supplementary Fig. 4a, 4b). As expected, kidney injury was significantly reduced with 4 weeks of succinate-enriched diet compared with EG-treated rats. Notably, the level of kidney injury was further reduced by the succinate treatment compared with the citric acid group, suggesting a greater efflux of oxalate from the kidney tissues, which was in keeping with the milder injury to the kidneys. By immunohistochemistry (IHC), intensities of CD44, IL-6, and OPN (Supplementary Fig. 5) were upregulated in tubules of EG-treated group than the control group. The administration of succinate has significantly inhibited the expression of CD44, IL-6, which was in keeping with the result of histological and functional analysis.

### Minimized transcriptomic changes of the EG-Induced kidney by succinate

**Fig. 6:**
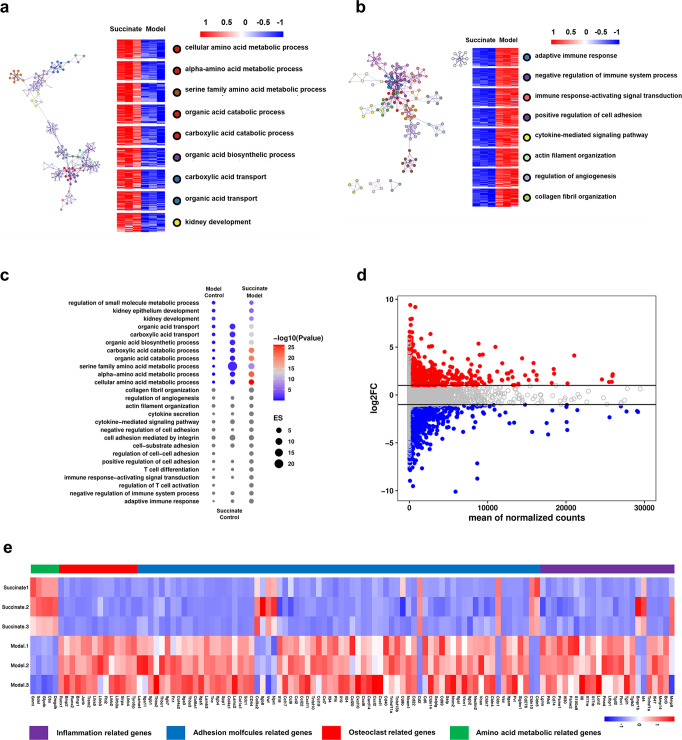
Reversal of EG-induced kidney damage by succinate as illustrated by RNA sequencing. **a**, **b** Gene ontology (GO) term enrichment analysis of the biological processes influenced by succinate and EG-treatment based on RNA-seq dataset. Up- (left) and downregulated processes (right) in the succinate treated group as compared with EG-treated model group. **c** Dot plot showing pairwise GSEA pathway comparison of the RNA-seq dataset of the succinate- and EG-treated model group and the corresponding controls. Blue and red dots respectively represented the down- and upregulated pathways. **d** A scatter plot showing expression fold change of DEGs between EG-treated model group and succinate group based on the RNA-seq dataset. **e** Heatmaps showing the expression of genes associated with inflammatory, apoptotic, and survival events in the rat kidney tissues from the EG-treated and succinate treated groups based on RNA-seq analysis. For (**a**–**e**), *n* = 3 rats in each group

The global impact of succinate on the rat model transcriptome reprogramming during crystal formation was measured based on RNA-seq of kidney tissues collected 4 weeks after the EG treatment. Pathway analysis and Q-PCR results suggested that, while differentially expressed genes (DEGs) in succinate group were significantly enriched for amino acid metabolism (Fig. 6a, c, Supplementary Fig. 7), while those in the succinate group were significantly enriched for immune response and cell-cell adhesion molecules (Fig. 6b, c, Supplementary Fig. 7) compared with the EG-treated model, which exerted a protective effect on the kidney injury. By RNA-seq analysis, the EG-treated group showed similar enrichment for pathways including pro-inflammatory, adhesion molecules and osteoclast differentiation cell, etc. in the model kidney compared with the controls (Supplementary Fig. 6a), but in the opposite direction in terms of amino acid metabolic pathway (Supplementary Fig. 6b).

Furthermore, the ameliorating function of succinate on kidney injury, including inflammatory response, adhesion molecules and osteoclast differentiation after EG treatment was largely reversed. This was verified by transcriptome-related heatmap analysis in which the changes in gene level in the model group were largely reversed by succinate. As shown, genes participating in the amino acid metabolism were mainly enriched in succinate group. By contrast, the EG-treated model showed a strong correlation with the kidney injury (i.e., gene induction), with enrichment of genes associated with cell-damage (Fig. 6d, e). Scatter plots and heatmaps analysis suggested that, among the DEGs, those of the control groups were obviously enriched for cellular amino acid metabolism, while those downregulated in the model group kidneys were obviously enriched for adhesion molecules and osteoclast differentiation compared with the EG-treated group (Supplementary Fig. 6d, e).

## Discussion

The leading events in the development of urinary stones include crystal nucleation, crystal growth, and crystal-cell adhesion.^38^ By targeting the interaction sites on the crystal surface, crystallization inhibitors may provide an alternative approach for reducing the rate of crystal growth. However, so far there has been no treatment for CaOx crystallization which could be used for the treatment of urinary stone disease.^22,39^ In this study, we have identified five potential stone intervention modifiers through metabolomics based on the theory of from stone (bladder stone) to stone (kidney stone). Among these, succinic, salicyluric acid and gentisic acid have shown the effect of crystal inhibition in vitro. Of note, when citric acid was used as a control for treating EG-induced rat kidney stone, succinic acid has shown excellent inhibition for renal calcium deposition in addition with a protective effect. Transcriptomic analysis has further shown that the protective effect of succinate was mainly through anti-inflammation, inhibition of cell adhesion and osteogenesis and regulation of amino acid metabolism.

Various modifiers of crystal growth, most commonly inhibitors, have been developed recently, which ranged from small molecules and peptides to proteins. Molecules with carboxylates, phosphates function groups have exert significant preventing function on crystal growth of COM.^38,40^ Based on this criteria and stringent screening, an in-depth analysis of non-target metabolomics data showed that taurine, suberic acid, succinic, salicyluric acid and gentisic acid with sulfate and carboxylate groups may interact with the crystal surface to affect its growth. To compare the in vitro efficacy of CaOx crystallization inhibitors is challenging for the difference of testing methods and parameters. In this study, we have combined DFT theoretical calculation and COM bulk crystallization tests to comprehensively assess the inhibitory effect of each modifier. Based on the mechanism that the crystal surface stain was imposed by inhibitor adsorption.^41,42^ The strain of modifier binding on COM (100) and (021) surfaces showed that gentisic acid, salicyluric acid and succinate showed a highest mean displacement (in Å) indicating that the crystal interactions are more energetic than taurine and suberic acid. We further calculated the binding ability of each molecule to Ca^2+^, which was in keeping with the trend of XPS results. The strength of the binding ability of small molecules to Ca^2+^ mainly depended on the electronegativity of oxygen in the C = O. The stronger the electronegativity, the stronger the binding energy to Ca^2+^. The results from crystallization assay indicated that succinic, salicyluric acid and gentisic acid could decrease the size and number of the crystals, which was consistent with previous studies of inhibitor for COM crystallization.^43,44^ During crystallization, succinic, salicyluric acid and gentisic acid can shift COM towards COD crystallization, which may confer a therapeutic effect as COD has lower affinity to epithelial cell membrane molecules and may thereby reduce intratubular retention.^11,45,46^

To explore the effect of crystal inhibitor on kidney stone in vivo, we have constructed an EG-induced rat model, where EG was used to induce excessive deposition of oxalate in renal tubules. Oxalate precipitated in renal tubules is the primary risk factors of CaOx kidney stones formation.^47^ Crystal inhibitors and citrate were applied to the rat models to assess their effect on the kidney stones. Among these, citrate is a kind of well-known inhibitor to inhibit kidney stone generation by combining with Ca^2+^ ions, leading to less free calcium to bind with oxalate.^48,49^ Kidney morphology changes in EG-induced rats were ameliorated by succinate application. We found that succinate dramatically reversed EG-induced CaOx deposition and reduced tubular cell injury and apoptosis. Cyto-protective effects of succinate were further confirmed in vivo by decreased serum creatinine levels. The protective mechanism of succinate on kidney CaOx deposition and injury was further investigated by RNAseq. A striking up-regulation of inflammation, cell adhesion and osteogenesis was caused by EG-induced of kidney. The anti-inflammatory and osteogenesis, succinate almost completely prevented the change in gene levels as well as up-regulation of amino acid metabolism-related genes. Succinate downregulated the expression levels of osteogenesis related genes, including IL-1, IL-6, and OPN, which is very close related to kidney injury and CaOx deposition in vivo.^50,51^ The decreased CaOx crystal adhesion by down-regulating the expression levels of CD68 and CD44 can provide an novel pathway to inhibit CaOx deposition in kidneys.^52^ The results warrant further investigation of the protective effect of succinate on kidney stones and modulation.^53^

In this study, we have identified a new crystal inhibitor succinate with exceptional efficacy, and shown its in vivo effect on CaOx deposition. Succinate shows promise as a effective therapy to prevent kidney stones. So far only a pilot rat kidney stone model study was conducted, aimed to verify the inhibitory effect on kidney CaOx deposition and injury. However, the effect of succinic on other urinary organs, in particular the bladder, has remained to be verified. At the same time, it will be interesting (and important) to confirm whether administration of succinate can also suppress the progression of kidney stones and alter the peripheral blood and urine metabolomics in other animal models. In the follow up study, a better understanding of the metabolic pathways of succinate in humans, optimal dosing regimen, tolerability are required before prospective clinical trials of succinate in this field. We are also very interested in the downstream targets of succinate, which is worthy to explore and may provide a path to advance its clinical translation.

## Materials and methods

### Materials

Calcium chloride (CaCl_2_, 97%), sodium oxalate (Na_2_C_2_O_4_, 99.5%), citric acid (C_6_H_8_O_7_, 99%), COM (CaC_2_O_4_·H_2_O), salicyluric (C_7_H_6_O_3_, 99%), succinate (C_4_H_6_O_4_, 99%), gentisic acid (C_9_H_9_NO_5_, 98%), taurine (C_2_H_7_NO_3_S, 99%), suberic acid (C_8_H_12_O_4_, 99%), sodium chloride (NaCl, AR) and EG (C_2_H_6_O_2_, 99.8%) were bought from Sigma Aldrich.

### Construction of a rabbit model for bladder stone

All experiments were approved by the Ethics Committee of Sichuan University (record number 2018190 A). Twenty male New Zealand white rabbits (2.5 ± 0.5 kg each) were randomly divided into the normal control and bladder stone model groups. After 2-week quarantine period, the experimental animals were fasted for 12 h before the surgery. Subsequently, they were anesthetized by an injection of pentobarbital sodium (2 mL/kg). An appropriately sized incision was made in the lower abdomen to expose the bladder. Then, partial full thickness bladder wall was removed, and the required posterior wall defect model was established. After sterilization, 1 mg/mL PC-SIS was fully sutured with soluble suture (5-0, Johnson, USA) 22 to obtain four suture lines (5-0 Monocryl) without obvious difference, so as to mark the defects of the back wall and provide support for subsequent analysis. The muscle and skin were sutured. After the operation, all the experimental animals were put back into the cage and fed freely. Penicillin was injected intramuscularly for three consecutive days. At different time points (1, 2, 3, and 4 weeks), rabbit urine was harvested by rabbit metabolic cage for non-targeted metabolomics analysis. Urine samples were collected in 1.5 mL EP tubes and centrifuged at a high speed condition for half an hour at 4 °C. The samples were kept at −80 °C environment. In addition, urine and blood samples were collected for urinary and blood calcium analysis.

### Ultrasonography of stone components

The bladder stone was detected by ultrasound at 2 and 4 week. And the warm sterile saline was injected into the bladder until maximum distension was achieved. Ultrasonography was performed with a ultrasound system (Philips, Best, Netherlands). The samples were collected, grind, and detected with a enhanced stone analysis system (Lanmode LIR-20) with wavelength range of 500–4000 cm^−1^ based on KBr pellet pressing method.

### Non-targeted metabolomic analysis

For non-targeted metabolomic analysis, 1 ml aliquot of rabbit urine sample was mixed with 1 ml of acetonitrile/methanol/water solution (2:2:1, v/v), vortex mixed, sonicated for 30 min, stand at −20 °C for 1 h, subsequently centrifuged with high speed for half an hour at 4 °C. Then dried under vacuum. For mass spectrometry, the dried sample was added 100 μL of acetonitrile (1:1, v/v) to resolve, and centrifuged at 15,000 × *g* for 20 min at 4 °C.

The non-targeted metabolomics were derived by using an ultrahigh property liquid chromatography system (UHPLC; Infinity LC; CA, USA) matched mass spectrometer (TOF 6600; AB Sciex, Concord, Canada). The urine metabolites were separated under an optimized condition at 4 °C with dose of 2 μL. The flow rate was 0.3 mL/min, the chromatographic separation program: 0–1 min, 85% B; 1–12 min, 85–65% B; 12–12.1 min, 65–40% B; 12.1–15 min, 40% B; 15–15.1 min, 40–85% B; 15.1–20 min, 8% B (A, 25 mM ammonium acetate and ammonium hydroxide in water; B, acetonitrile). During UHPLC-MS analysis, the control temperature is 4 °C. During mass spectrometry analysis, ESI source was selected and different modes of detection were conducted at the source temperature of 600 °C.

After the original data is determined, the ProteoWizard tool is used to convert it to obtain data in the. mzML format. Then, the XCMS is used to perform peak alignment and correction operations, and the information related to the peak area is extracted. For XCMS extract, remove the corresponding ion peak when it is judged that the missing value is >50%. In pattern recognition, SIMCA-P 14.1 software is applied, and Pareto scaling method is used to preprocess the data, and then statistical analysis is carried out, mainly including PCA and PLS-DA analysis. The PLS-DA method is mainly used to determine R2 and Q2 quality, as well as variable importance (VIP). VIP mainly describes the changes of pathological stimulus reactions explained by specific metabolites. When judging the metabolite with significant difference, the set judgment criteria were set as VIP > 1 and *P* < 0.05. Differentially expressed metabolites were normalized to obtain clusters with similar expression patterns. Heat map was derived by clustering differentially expressed metabolites based on R software. The KEGG analysis was carried out on hypergeometric test, the pathways with *P* < 0.05 were considered as significant.

### COM bulk crystallization

CaCl_2_ and Na_2_C_2_O_4_ were respectively dissolved in deionized water to form a stock solution of 10 mM. Bulk crystallization of 10-mL reaction system was performed in clean glass vials. Firstly, 150 mM NaCl aqueous solution was added to the vial, then 0.7 mL CaCl_2_ solution were added while mixing evenly. Subsequently, the glass vials were placed to incubator at 60 °C for 1 h before dropping 0.7 mL of Na_2_C_2_O_4_ stock solution. Crystal inhibitors, e.g., taurine, suberic acid, succinate, salicyluric and gentisic acid, were added into the reaction system at an appropriate quantity before Na_2_C_2_O_4_ addition and the final solution contains 1.5 mM crystal inhibitor. To collect the crystals, clean glass slides were placed in the bottom of the vial. After incubating at 60 °C for 3 days, the slides were removed and following washing as well as drying at 25 °C for subsequent characterization.

### Characterization of COM crystallization

The morphology and density of crystals were first analyzed with an optical microscope (Ti2-U, Nikon, Japan). The area and number of crystals in at least five fields were estimated with Image J software. Moreover, the ratio between COM and calcium oxalate dihydrate (COD) was analyzed by counting all crystals. The morphology of crystals on the glass slide was further examined by SEM (EVO, ZEISS, Germany) after gold sputtering treatment. In particular, two different types of calcium oxalate, namely COM and COD, were observed and photographed. To further evaluate the effect of various crystal inhibitors on CaOx crystallization, atomic force microscopy (AFM, Asylum Research, USA) was carried out to examine topographical images of the crystal (001) surface, particularly the etching phenomenon. The images were collected using the tips with 60 N/m of spring constants under the tapping mode (scan range: 2 μm; scan rate: 0.5 Hz).

The structure of the crystals was validated with XPS (Kratos, UK) and Fourier transform infrared spectroscopy (FTIR, INVENIO R, Switzerland). The FTIR spectrum of various COM crystals was measured with wavelength scope of 1000–4000 cm^−1^. As to XPS, the C 1s, N 1s and O 1s spectrum of various COM crystals, taurine, suberic acid, succinate, salicyluric and gentisic acid were fit using CasaXPS software.

### Density functional theory (DFT) calculation

The density functional theory (DFT) was used to explore the interactions between various crystal inhibitors and CaC_2_O_4_ surface theoretically.^42^ The generalized gradient approximation (GGA) functional was applied. The Monkhorst-Pack method was applied in the Brillouin zone sampling process. The convergence standard of energy was 10^−5 ^eV/atom.

The binding energies of the crystal inhibitor molecules, including succinic, taurine, salicyluric, suberic acid and gentisic acids, on the CaC_2_O_4_ surfaces were calculated. The CaC_2_O_4_ (100) and (021) surfaces were built by the 2 × 2 and 2 × 1 supercells, respectively. A vacuum space of 15 Å was applied to preventing interactions. The DFT-D2 method was applied to calculate the vdW interactions between the crystal inhibitor molecules and CaC_2_O_4_ surface. While the formula of binding energies (*E*_b_) were as follow:

$$E_b = E_{\left( {\rm{Molecule}}\right) - \left.{\rm{CaC}}_2{\rm{O}}_{4}\right)} - (E_{\rm{Molecule}} + E_{{\rm{CaC}}_{2}{\rm{O}}_{4}})$$

where $E_{\left( {\rm{Molecule}} - {\rm{CaC}}_{2}{\rm{O}}_4\right)}$, and $E_{{\rm{CaC}}_2{\rm{O}}_4}$ refer to the DFT energies of molecule adsorbed CaC_2_O_4_ surface, the clean CaC_2_O_4_ surface, respectively.

This study have geometrically compared the frozen and relaxed structures of CaC_2_O_4_ surface with or without the presence of adsorbates (growth inhibitor), as referred from our previous work. A distance metric was used to quantify the displacement of relaxed atoms on the surface of CaC_2_O_4_ crystal. The average displacement *δ* can be calculated by the following formula:

$$\delta = \frac{{\mathop {\sum}\nolimits_{i = 1}^{N_{{{{\mathrm{atoms}}}}}} {\sqrt {\left( {x_{i,\,{{{\mathrm{frozen}}}}} - x_{i,\,{{{\mathrm{relaxed}}}}}} \right)^2 + \left( {y_{i,\,{{{\mathrm{frozen}}}}} - y_{i,\,{{{\mathrm{relaxed}}}}}} \right)^2 + \left( {z_{i,\,{{{\mathrm{frozen}}}}} - z_{i,\,{{{\mathrm{relaxed}}}}}} \right)^2} } }}{{N_{{{{\mathrm{atoms}}}}}}}$$

In which *N*_atoms_ refer to the atoms number relaxed on CaC_2_O_4_ plane surface.

Furthermore, the average displacement and binding energies between Ca^2+^ and the crystal inhibitor molecules (i.e., taurine, suberic acid, succinate, salicyluric, gentisic acid) were derived with a similar method.

### Construction of kidney stone model

Male SD rats bought from Chengdu Dossy Animals Factory, and were classified into the blank control group, kidney stone model group, and treatment groups (citric acid, taurine, suberic acid, succinate, salicyluric and gentisic acid), with each group containing at least five rats. Ethylene glycol was used for constructing the kidney stone model with the SD rats.^54^ Specifically, the model was built by continuously feeding drinking water containing 1% v/v EG for 28 days, whilst the control group was fed by drinking water only. During the modeling, treatment was performed with citric acid, taurine, suberic acid, succinate, salicyluric, and gentisic acid, respectively. The inhibiting dose (200 mg/kg) used for the rat kidney stone model was calculated based on the clinical therapeutic concentration of citric acid (the effective component of uralyt-U) which was converted for rats based on the body surface area. Subsequently, the rats were treated with aqueous solutions of taurine, suberic acid, succinate, salicyluric, and gentisic acid by daily gavage for 28 days. All rats were alive throughout the experiment. The initial weight of each rat was recorded, and the amount of drug gavage was adjusted according to its daily weight.

### Tissue collection and histopathological analysis

The rats were anesthetized, fixed in the supine position, with the heart fully exposed. 3 mL of blood was collected from the right ventricle with a disposable vacuum blood collection tube, and the kidneys were harvested. The samples were left at 25 °C for 15 min and centrifuged for 15 min. 1 mL of supernatant was aspirated for biochemical assays. The morphology of the kidneys was recorded by photography. And each kidney was weighed and recorded. The kidneys were then stored in 4% paraformaldehyde fixative solution or frozen for subsequent analysis.

The kidney samples were fixed in 10% formalin solution for paraffin embedding, and sectioned at a thickness of 10 μm for staining. Crystal formation were detected based on the Von Kossa staining. Crystal deposits were analyzed by optical microphotography and Image J software. Kidney sections were subjected to (PAS staining, and tubular injury was scored by PaIIer’s method: tubular dilatation and flat epithelial cells (1 point); tubular cast (2 points); necrotic and exfoliated cells in the tubular lumen with no debris (1 point); epithelial cell pyknosis (1 point).^55^

For immunohistochemistry (IHC), the kidney sections were incubated in xylol, gradient ethanol and thereafter in 3% H_2_O_2_ for blocking the activity of endogenous peroxidase. The CD44 (ab189524, Abcam), IL-6 (ab9324, Abcam) and OPN (ab11503, Abcam) antibodies were incubated at 4 °C overnight. The section was rinsed in PBS and incubated with HRP-conjugated secondary antibodies at 1: 200 dilution for 1 h at 37 °C, exposed to DAB for the detection of conjugated HRP. And the images were assessed with Image J tool.

### Bulk RNA-sequencing and differential expression analysis

Four weeks postoperatively, rat kidneys were collected from the control group, modeling group and succinic acid group (*n* = 3). In the process of extracting total RNA, the RNeasy kit (Qiagen, Germany) was used to preserve the obtained RNA at ultra-low temperature. According to the corresponding instructions, TruSeq was synthesized through the corresponding RNA kit (Illumina, USA). In this synthesis experiment, firstly, the mRNA containing poly A was purified by magnetic beads. Then, based on the corresponding kit scheme, the mRNA was randomly fragmented to obtain double stranded cDNA. Then, the end repair and dA tail treatment were carried out to provide support for subsequent operations. The product was purified and enriched by PCR to obtain the required cDNA library. Qubit is applied to the quantitative analysis of the purified library ® 2.0 Fluorometer (Life Tech, USA) and 2100 analyzer (Agilent, USA) was used to check the library, and then calculates and analyzes the molar concentration. After diluting the purified library, clusters were generated by cBot and tested. NovaSeq 6000 (Illumina, USA) was used in the sequencing process.

Use Hisat2 to map the paired terminal sequence file (fastq) to the Ensembl reference genome, and then convert the output SAM file to obtain the BAM file. During sorting, SAMtools 1.3.1 is applied to convert the mapped readings to form the BAM file, which provides support for subsequent FPKM analysis. The genes were analyzed by StringTie1.33b tool. Differential expression analysis (DEGs) for mRNA was conducted based on R package edgeR, and genes with fold change of over 1.5 and *P* < 0.05 were regarded as DEGs. Volcanic plot was drawn with the software package EnhancedVolcano. The standardized gene expression of FPKM was obtained by processing with clustering tools in R package, and the gene clusters with similar expression were determined on this basis. In the heat map, the gene expression data is normalized by the formula *y* = (*x*-*α*)/*λ*, in which *x* refer to the actual expression, *λ* corresponding to the variance of samples. Metascape online tool is used for GO enrichment analysis. The data set of DEG was analyzed by RNA seq, and its distribution was determined. When *P* is <0.05, the pathway is considered significant

### Quantification of gene expression by RT- PCR (Q-PCR)

Total RNA was isolated from the kidney samples by TRzol reagent, and cDNA was prepared through reverse transcription based on PrimeScript™ kit (Takara, Japan). Q-PCR was performed on a Lightcycler 96 system (Roche, Switzerland) to detect the gene expression at the mRNA level. The primers can be seen appendix Supplementary Table 6. The thermal cycles program: 95 °C for 30 s; 40 cycles of 95 °C for 10 s and 60 °C for 30 s. The relative level of gene expression by Q-PCR was calculated with a 2^−ΔΔ*Ct*^ method.

### Statistical analysis

All data were expressed as mean ± SD. Statistical difference were checked with independent *t*-test or one-way analysis of variance with Tukey’s test using SPSS 11.0. The significant threshold was set as 0.05.

## Supplementary information

Supplementary information

## Data Availability

All data in this research were presented within the paper and/or the Supplementary Information. The RNA-seq raw data were deposited in the SRA database with access code PRJNA874063 and the original non-targeted metabolomic data have been deposited in the MetaboLights with accession number MTBLS5921.
