# Supplementary material for: Application of metabolomics in urolithiasis: the discovery and usage of succinate
Source: Signal Transduct Target Ther. 2023 Jan 21;8:41. doi: 10.1038/s41392-023-01311-z (PMC9867757; doi:10.1038/s41392-023-01311-z)
Supplement: Supplementary file 1 — Supplementary information [file 41392_2023_1311_MOESM1_ESM.docx]

Supplementary Materials for

Application of Metabolomics in Urolithiasis: The Discovery and Usage of Succinate

Xiu-zhen Zhang^a^, Xiong-xin Lei^a^, Yan-lin Jiang^a^, Long-mei Zhao^a^, Chen-yu Zou^a^, Yun-Jin Bai^b^, Ya-xing Li^b^, Rui Wang^a^, Qian-jin Li^a^, Qiu-zhu Chen^a^, Ming-hui Fan^a^, Yu-ting Song^a^, Wen-qian Zhang^a^, Yi Zhang^b^, Jesse Li-Ling^a,c^, Hui-qi Xie^a,*^

Correspondence to: [xiehuiqi@scu.edu.cn](mailto:xiehuiqi@scu.edu.cn)

**This PDF file includes:**

Figures S1 to S7

Tables S1 to S6

Movies S1

**
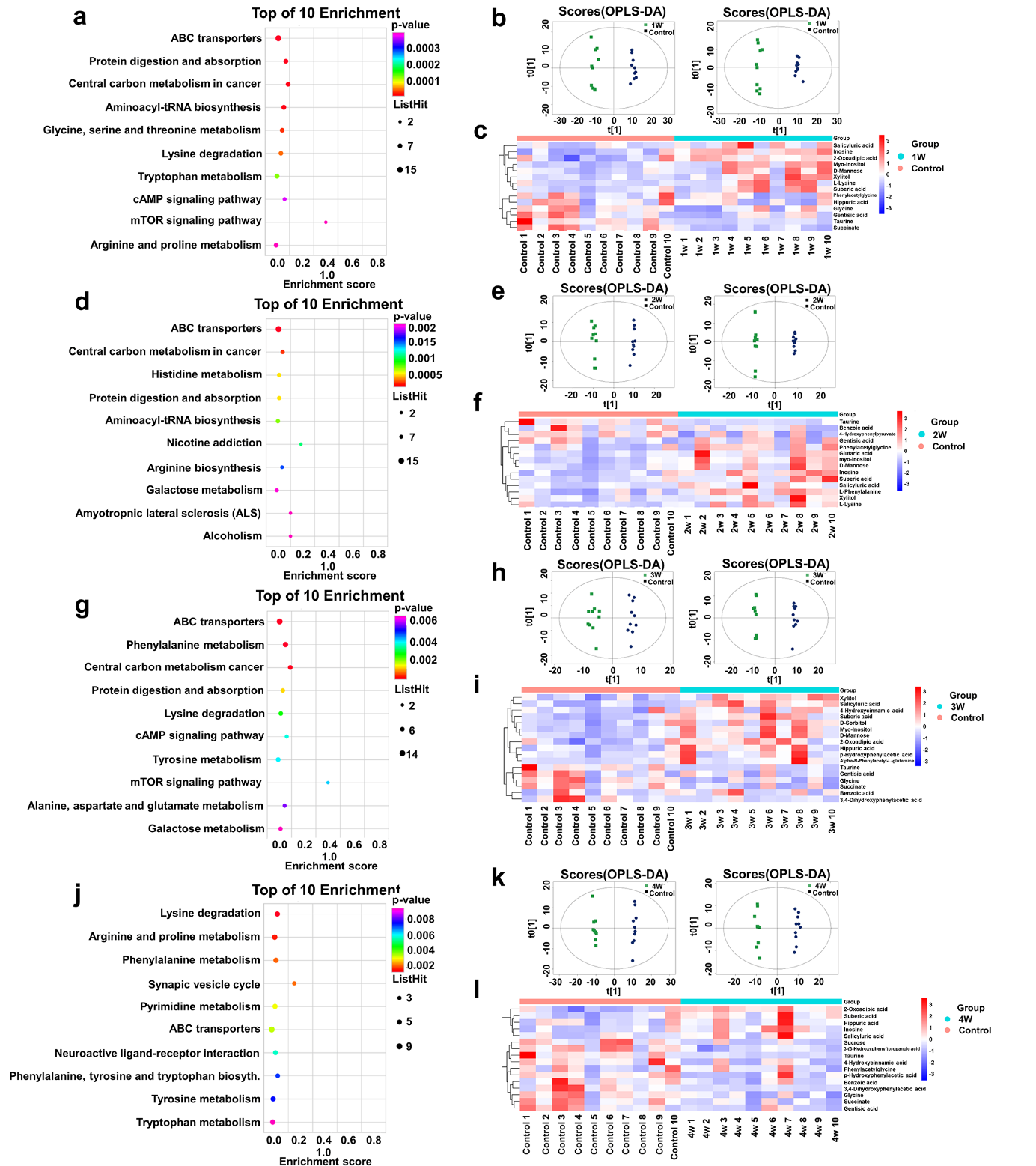
**

**Supplementary Figure 1.** Metabolomic analysis of the control and model groups at various time points. a, d, g, j, KEGG pathway enrichment analysis of the control and model groups at various time points. b, e, h, k, Partial least squares discriminant analysis (PLS-DA) showing the positive and negative ions of the control and model distribution based on the metabolite profiles of urine samples. c, f, i, l, Heatmap of the ABC transporters, lysine degradation, phenylalanine metabolism and tyrosine metabolism pathways. **P* < 0.05. For (a ~ e), n = 10 rabbits in each group


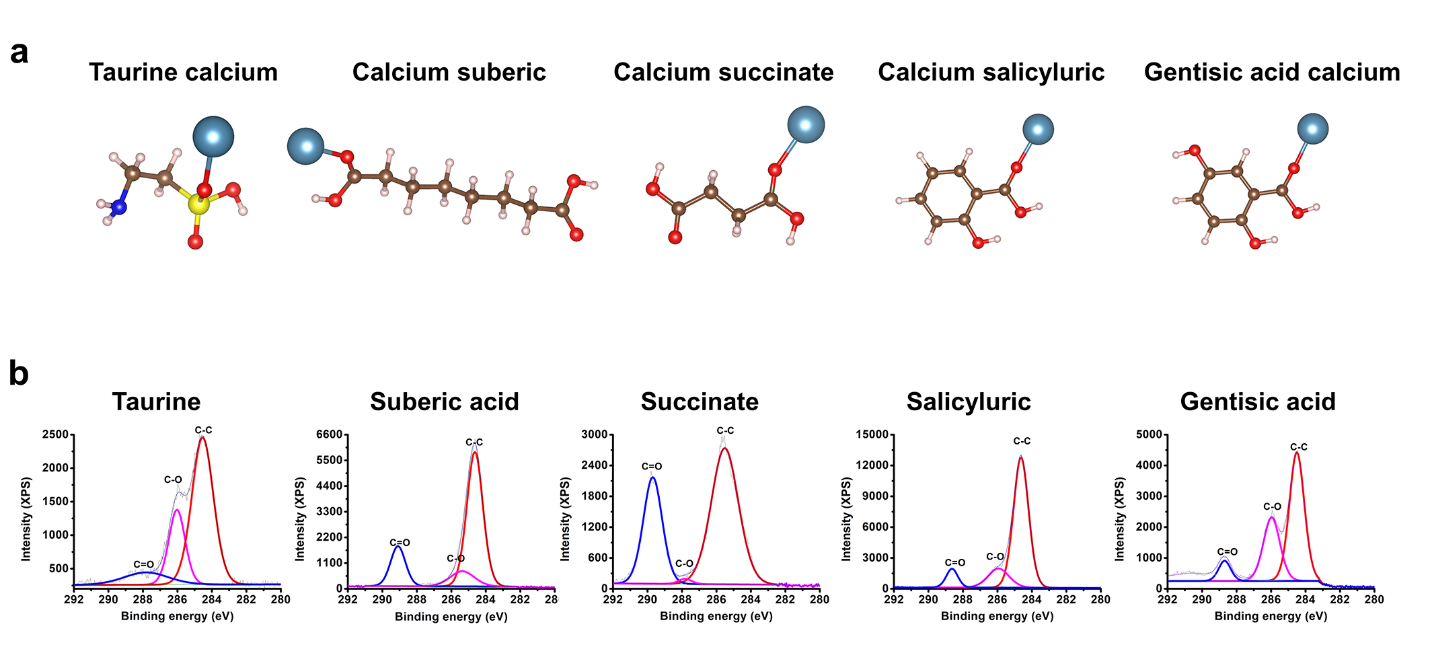


**Supplementary Figure 2.** Binding energy of the modifiers on the Ca^2+^. a, Optimized structure of Ca^2+^ on the modifiers. The light pink, light-blue, brown, blue, yellow and red balls denote H, Ca, C, N, S and O atoms, respectively. b, X-ray photoelectron spectra (XPS) of taurine, suberic acid, succinic, salicyluric and gentisic acids. The fitted peaks in blue, pink and red correspond to the C1s peaks of C=O, C-O and C-C, respectively


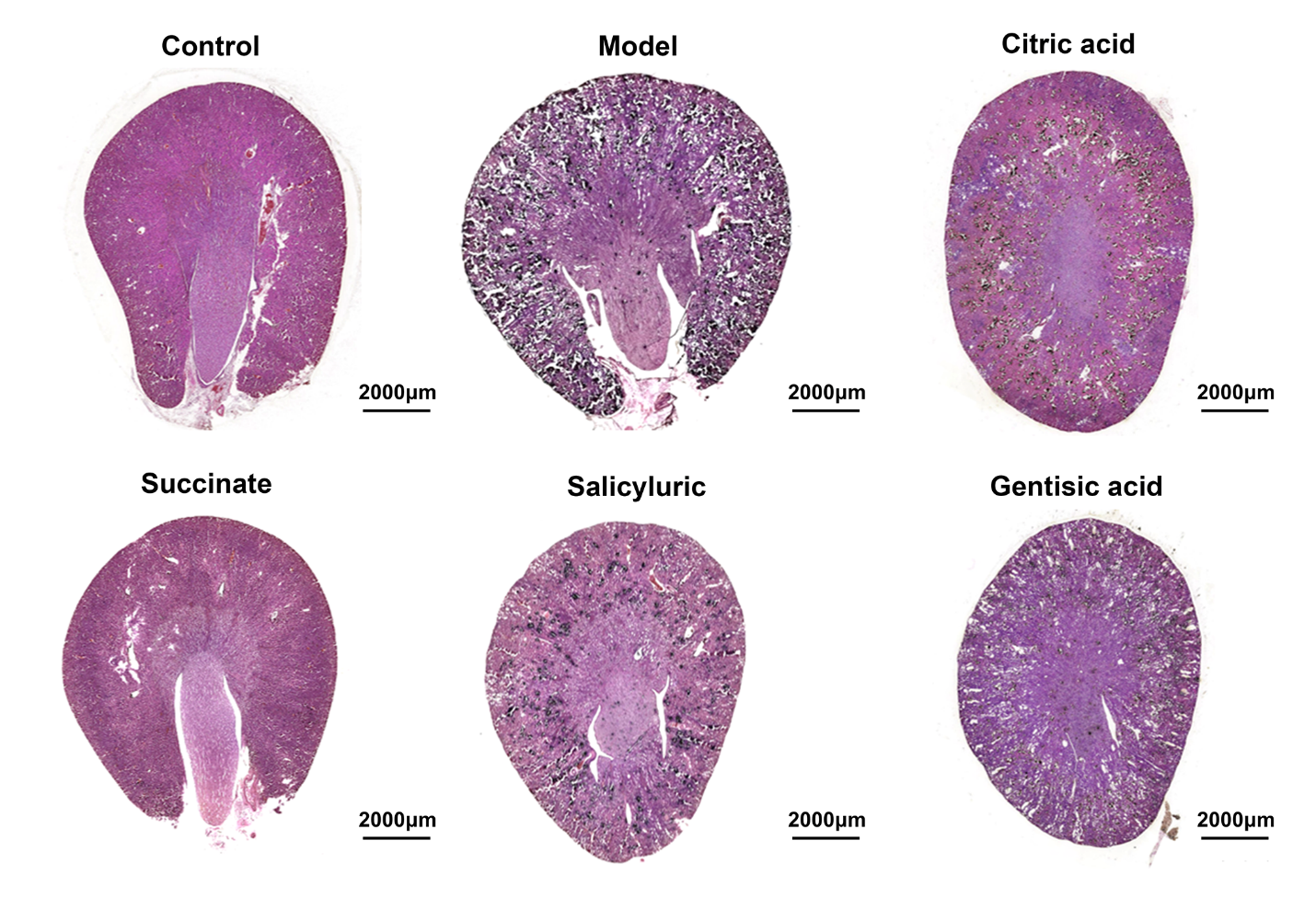


**Supplementary Figure 3.** Representative images of Von Kossa staining of kidney sections

(scale bar: 2000 μm)

**
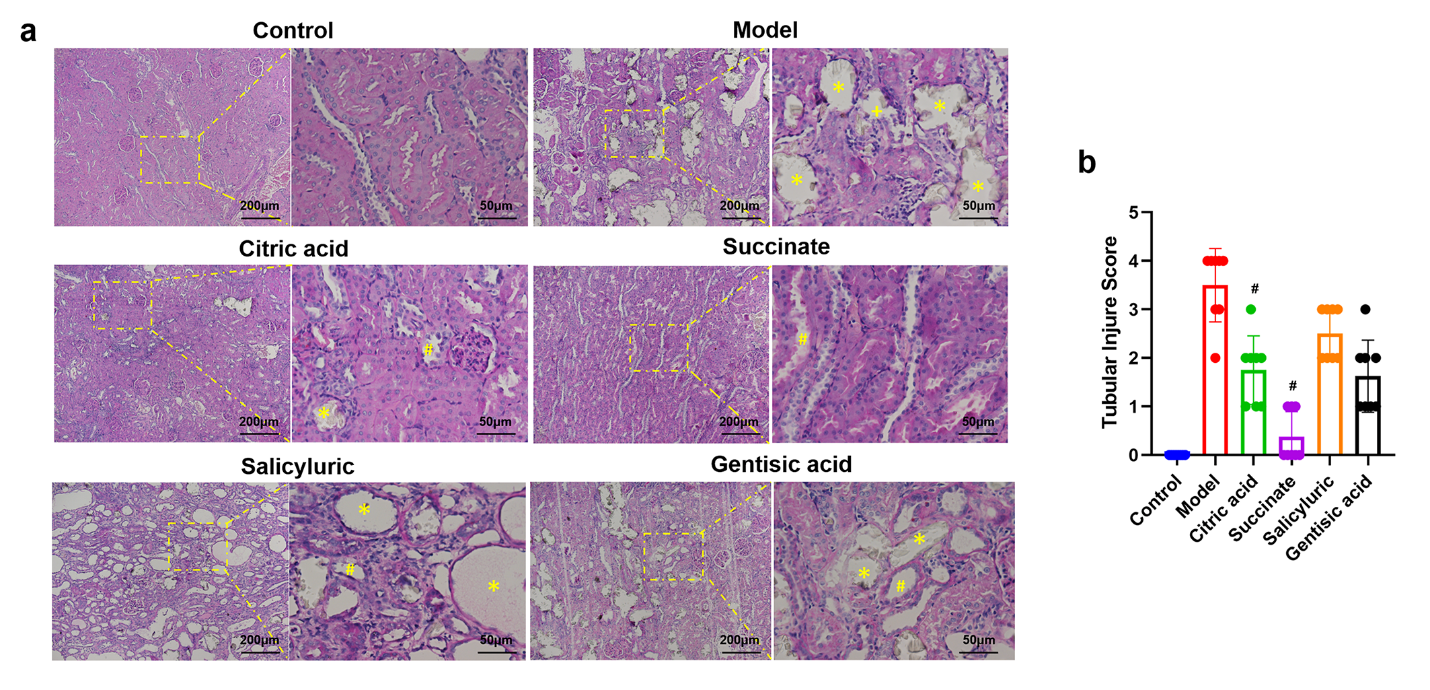
**

**Supplementary Figure 4.** Kidney sections were subjected to PAS staining and scored for kidney injury. a, PAS staining of the kidney sections with tubular injury highlighted (*: necrotic areas; #: dilated tubules; +: tubular casts; scale bars: 200 µm & 50 μm). b, Scores of kidney tubular injury, # *P* < 0.05 compared with the model group

**
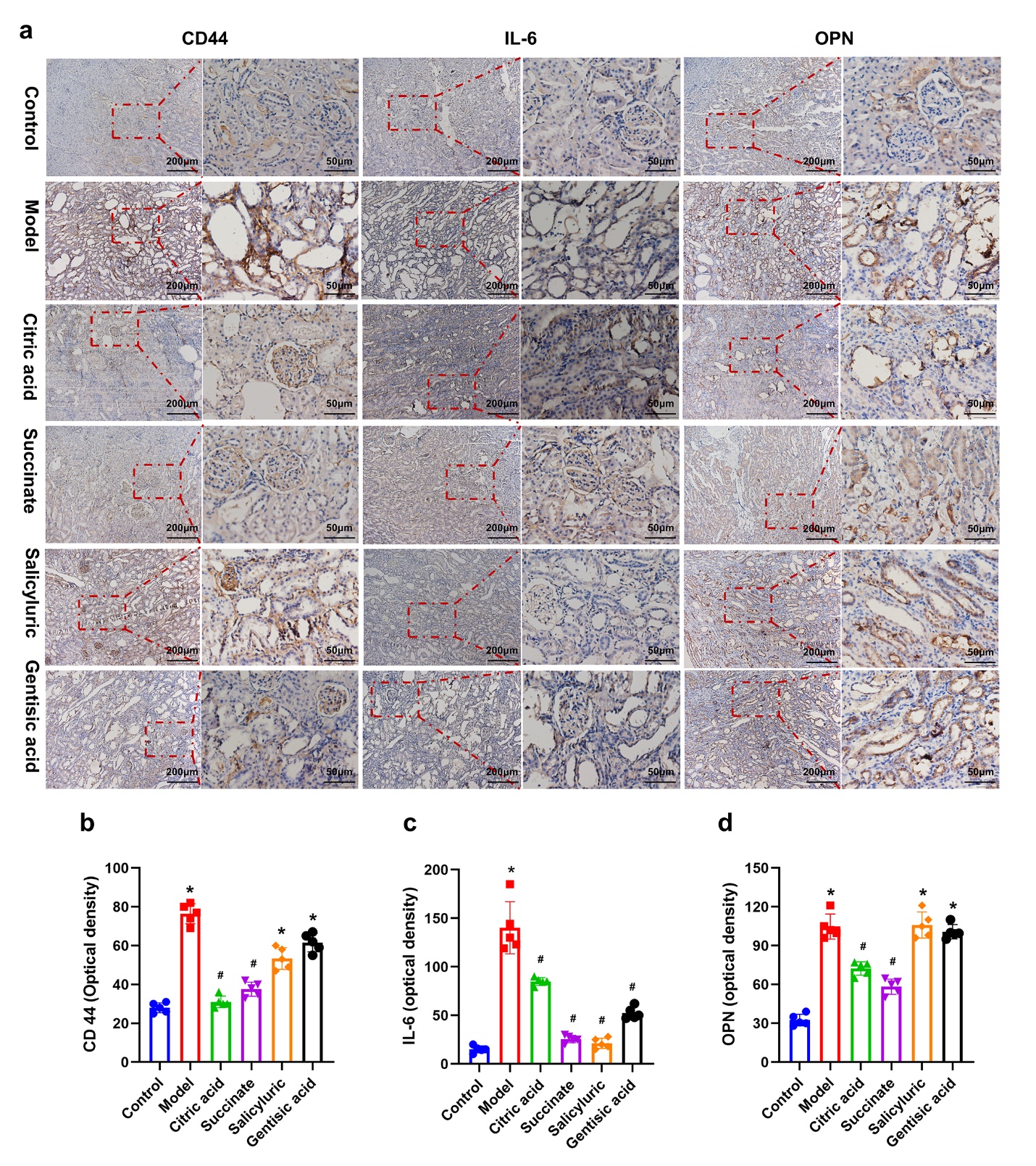
**

**Supplementary Figure 5.** Immunohistochemistry staining and semi-quantitative analysis a, immunohistochemistry staining of CD44, IL-6 and OPN in kidney tissues from each group of rats (scale bars: 200 µm & 50 μm). b ~ d, Sections of rat kidney subjected to immunohistochemistry staining for CD44, IL-6 and OPN. Positive areas as indicated were quantified, * *P* < 0.05 compared with the control group; # *P* < 0.05 compared with the model group

**
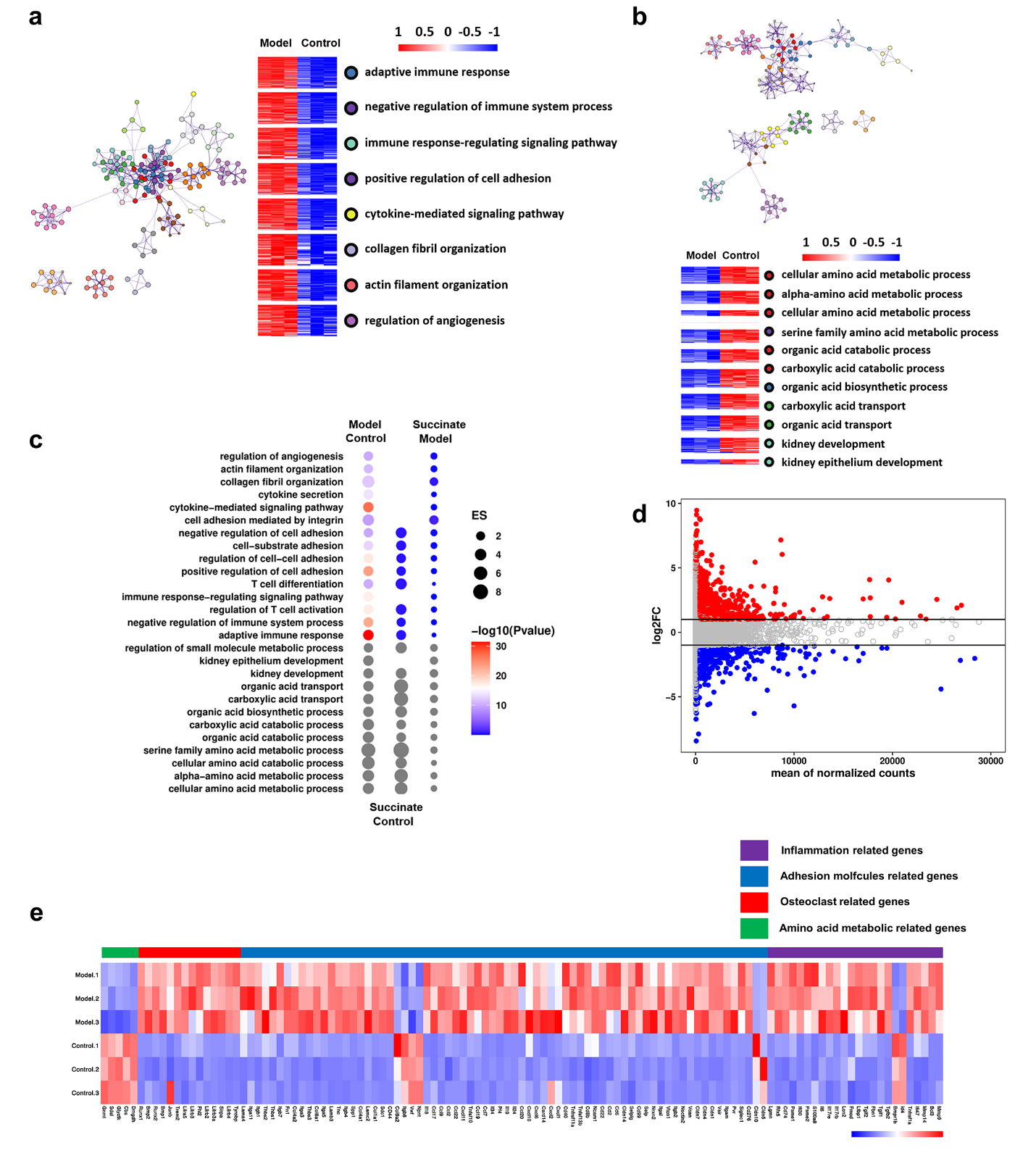
**

**Supplementary Figure 6.** RNA-seq data of the normal control and the rat model. a,b, Gene ontology (GO) term enrichment analysis of biological processes influenced by the EG treatment based on the RNA-seq dataset. Up-regulated (left) and down-regulated processes (right) from the succinate group compared to EG-treated model group are shown. c, Dot plot showing pairwise GSEA pathway comparison of RNA-seq dataset of the succinate group, EG-treated model group and the controls. Blue and red dots respectively indicated down- and up-regulated pathways. d, A scatter plot representing expression fold change of differentially expressed genes (DEGs) between the EG-treated model and the control groups based on the RNA-seq dataset. e, Heat maps of mRNA DEGs related pathways between the EG-treated model and control groups detected by the RNA-seq assay. For (a ~ e), n = 3 rats in each group


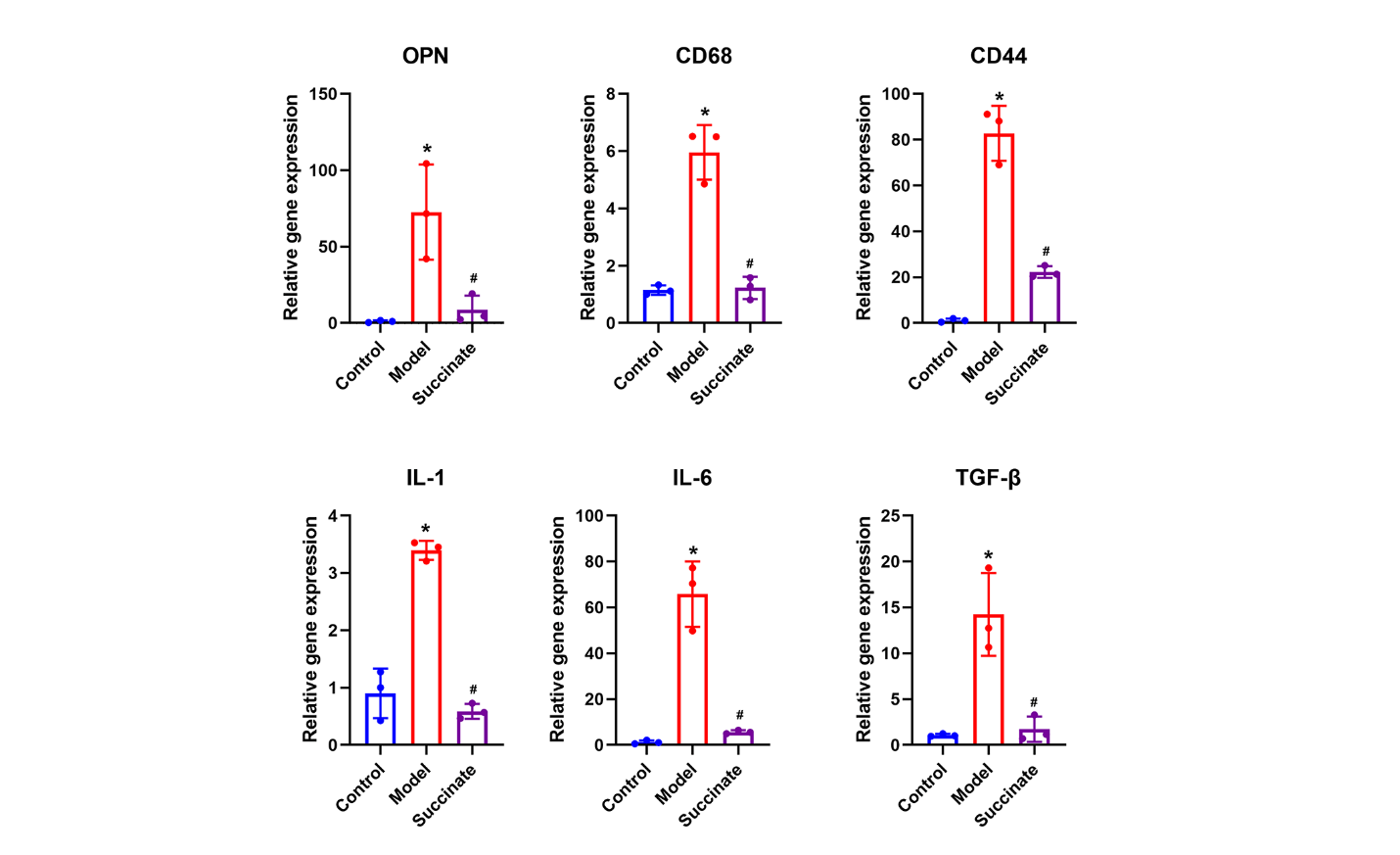


**Supplementary Figure 7.** Q-PCR analysis of the mRNA expression of genes associated with osteogenesis, inflammation and cell adhesion after the EG-induction and succinate treatment, * *P* < 0.05 compared with the control group; # *P* < 0.05 compared with the model group

**Supplementary Table**

**Supplementary Table 1** Size of the bladder stones

| **Group** | **Model 1** | **Model 2** | **Model 3** | **Model 4** |
| --- | --- | --- | --- | --- |
| Crosswise 2W | 1.60 cm | 1.32 cm | 1.58 cm | 1.24 cm |
| Lengthways 2W | 1.03 cm | 1.12 cm | 0.80 cm | 1.13 cm |
| Crosswise 4W | None | 1.39 cm | None | None |
| Lengthways 2W | None | 1.18 cm | None | None |

**Supplementary Table 2** Binding energy (E_b_, eV) of molecules adsorption on the CaC_2_O_4_ surface

| **Binding energy on COM (E_b_, eV)** | **(100)** | **(021)** |
| --- | --- | --- |
| Succinic, C_4_H_6_O_4_ | 2.99 | - 0.78 |
| Taurine, C_2_H_7_NO_3_S | 2.42 | - 0.41 |
| Salicylic, C_7_H_6_O_3_ | 9.38 | - 0.51 |
| Suberic acid, C_8_H_14_O_4_ | 2.95 | - 0.91 |
| Gentisic acid, C_7_H_6_O_4_ | 10.17 | - 1.68 |

Positive values represent that the binding processes are exothermic

**Supplementary Table 3** Mean displacement (in Å) of surface atoms after molecules binding on CaC_2_O_4_ (100) plane

|  | **Average displacement on COM (100), δ (values in Å)** | **Average displacement on COM (021), δ (values in Å)** |
| --- | --- | --- |
| Succinic, C_4_H_6_O_4_ | 0.249 | 0.198 |
| Taurine, C_2_H_7_NO_3_S | 0.211 | 0.099 |
| Salicylic, C_7_H_6_O_3_ | 0.398 | 0.165 |
| Suberic acid, C_8_H_14_O_4_ | 0.244 | 0.142 |
| Gentisic acid, C_7_H_6_O_4_ | 0.418 | 0.206 |

**Supplementary Table 4** Binding energy (E_b_, eV) of molecules on Ca^2+^ ion

| **Binding energies on Ca^2+^** | **(E_b_, eV)** |
| --- | --- |
| Succinic, C_4_H_6_O_4_ | - 0.451 |
| Taurine, C_2_H_7_NO_3_S | - 0.101 |
| Salicylic, C_7_H_6_O_3_ | - 0.586 |
| Suberic acid, C_8_H_14_O_4_ | - 0.300 |
| Gentisic acid, C_7_H_6_O_4_ | - 0.594 |

**Supplementary Table 5** Positional change of C=O

| **Binding energies** | **C=O** | **C=O (complex Ca^2+^)** | **ΔC=O** |
| --- | --- | --- | --- |
| Succinic, C_4_H_6_O_4_ | 289.0965 | 288.5226 | 0.5739 |
| Taurine, C_2_H_7_NO_3_S | 288.4819 | 288.1637 | 0.1797 |
| Salicylic, C_7_H_6_O_3_ | 288.6938 | 288.0697 | 0.6241 |
| Suberic acid, C_8_H_14_O_4_ | 288.5967 | 288.8994 | -0.3027 |
| Gentisic acid, C_7_H_6_O_4_ | 287.8218 | 288.6365 | -0.8147 |

**Supplementary Table 6** Primers for qPCR analysis

| **Species** | **Gene** | **Forward primer (5’ to 3’)** | **Reverse primer (5’ to 3’)** |
| --- | --- | --- | --- |
| Rat | OPN | GAGACCATGCAGAGAGCGAGGA | GGGCGATTGGAGTCAAAACG |
| Rat | IL-6 | AGTTGCCTTCTTGGGACTGATGT | GGTCTGTTGTGGGTGGTATCCTC |
| Rat | CD44 | CCACAGCCTCCTTTCAATAACCAT | TGCCATTCATTCTCAAACCACTTC |
| Rat | TGFβ | CTAATGGTGGACCGCAACAAC | CACTGCTTCCCGAATGTCTGA |
| Rat | CD68 | TCAAACAGGACCGACATCAGA | ATTGCTGGAGAAAGAACTATGCT |
| Rat | IL-1 | AGGAGAGACAAGCAACGACA | CTTTTCCATCTTCTTCTTTGGGTAT |
| Rat | Gapdh | TCTCTGCTCCTCCCTGTTC | ACACCGACCTTCACCATCT |

**Supplementary Movies 1**
